# Identifying Priorities for Enhancing Village Health Volunteer's Mental Health Recovery Practices in Thai Rural Communities: A Nominal Group Technique Study

**DOI:** 10.1111/hex.70455

**Published:** 2025-10-03

**Authors:** Chonmanan Khanthavudh, Annmarie Grealish, Vasiliki Tzouvara, Mary Leamy

**Affiliations:** ^1^ Florence Nightingale Faculty of Nursing, Midwifery & Palliative Care King's College London London UK; ^2^ Ramathibodi School of Nursing, Faculty of Medicine Ramathibodi Hospital Mahidol University Bangkok Thailand; ^3^ School of Nursing and Midwifery, Health Research Institute University of Limerick Limerick Ireland

**Keywords:** Asia, community health worker, community mental health, mental health recovery, Nominal Group Technique, recovery‐oriented practice, Village Health Volunteer

## Abstract

**Background:**

World Health Organization (WHO) and Thailand's national policy both advocate for recovery‐oriented, community‐based mental healthcare. Village Health Volunteers (VHVs) in Thailand have limited involvement in mental health services despite their pivotal role in Thai primary healthcare, especially in rural settings. This study aims to engage stakeholders to identify and prioritise key areas for VHVs' role expansion, stigma reduction, training needs, and common mental health conditions, thereby enhancing VHVs' contributions to recovery‐oriented mental healthcare in rural Thai communities.

**Methods:**

The study utilised the Nominal Group Technique (NGT). Eight VHVs, six individuals with mental health challenges and caregivers, and four healthcare professionals (HCPs), from three rural sub‐districts in Northern Thailand, were purposively and conveniently recruited. Three NGT groups were formed: in‐person for service providers (VHVs and HCPs) and service users/caregivers, and online for HCPs. Through structured stages of idea generation, sharing, discussion and ranking, participants identified their top five priorities. Ranked priorities were synthesised, and transcripts were analysed using content analysis.

**Results:**

Eighteen participants attended one of three NGT groups. Collectively, these groups generated 94 ideas in response to four questions. Four themes were identified: (1) Expansion of VHV's mental health role, including vocational support, family support, emotional support and community reintegration; (2) Stigma reduction, focusing on changing attitudes, implementing a buddy system and enhancing mental health literacy; (3) Training needs including training related to stigma reduction, improving communication skills and providing mental health awareness education; and (4) Common mental health conditions, including psychosis and depression.

**Conclusion:**

The prioritisation among the three groups varies. High priorities include vocational support, family support, community reintegration and counselling skills. Addressing stigma is a starting point and can be achieved through increased awareness and literacy. Future research should focus on tailored stigma interventions and trainings to support VHVs in providing effective, recovery‐oriented care in these communities.

**Patient or Public Contribution:**

Six patient and public involvement (PPI) advisors participated in the study, comprising two VHVs, one mental health nurse, one caregiver and one peer support worker, to ensure research relevance and applicability. The PPI reviewed the Thai NGT questions to assess the appropriateness of language, particularly in relation to mental health and stigma, leading to minor modifications in wording. Additionally, two VHVs and one caregiver assisted in piloting the questions to evaluate their validity and appropriateness and offered feedback on the procedure, content and timing. They recommended using probing questions to elicit more detailed responses and ensuring concise content to maintain participant engagement.

## 1 Introduction

The World Health Organization (WHO) [1] advocates for community‐based mental healthcare to improve accessibility, reduce stigma and support reintegration. It highlights the role of community health workers (CHWs) in expanding care access, reducing inequities and advancing Universal Health Coverage. This also contributes to achieving the Sustainable Development Goals (SDGs), particularly in Low‐ and Middle‐Income Countries (LMICs) [2, 3, 4]. According to the International Labour Organization (ILO), CHWs are individuals who deliver health education, make referrals and provide support to communities, families and individuals in both preventive and curative care [4]. They play an important role in connecting communities, particularly those facing barriers to care, with health, social and community services [4]. Yet, the term is used ambiguously. CHWs, whether paid or volunteer, are typically non‐professionals with limited training [2, 5].

CHWs have become indispensable to primary healthcare systems worldwide, offering a comprehensive range of services, including health promotion, disease prevention, treatment and rehabilitation. Their role is increasingly recognised for its potential to reduce the burden of non‐communicable diseases, enhance maternal and child health, manage infectious diseases, and strengthen primary healthcare systems overall [3, 6]. Additionally, CHWs play a pivotal role in improving health outcomes within underserved and marginalised communities by leveraging local resources and fostering community engagement [2, 5].

In Thailand, an upper‐middle‐income country in Southeast Asia, CHWs are referred to as Village Health Volunteers (VHVs) or *Or Sor Mor*, a designation established through national policy. The VHV model was introduced in the 1970s and has since expanded to include over 1 million non‐professional volunteers who play a critical role in supporting local healthcare services [7, 8]. Each VHV is typically responsible for overseeing 10–20 households and assists in various public health initiatives under the guidance of local health professionals [2, 5]. VHVs do not need formal educational qualifications before selection but receive comprehensive training in various areas such as health management, infectious disease control, non‐communicable disease management and mental health [8]. They receive a modest remuneration of 2000 baht (44 GBP) per month. VHVs play a crucial role in Thai public health, contributing to dengue haemorrhagic fever prevention [9], HIV control [10], children's oral health promotion [11], avian flu management [12] and Covid‐19 surveillance [13]. However, evidence of their significant role in mental health is limited.

Mental health services worldwide are increasingly designed to support personal recovery, helping individuals rebuild their lives and regain a sense of purpose despite challenges posed by the illness [14, 15]. WHO also highlights the need for mental healthcare that focuses on people's needs, respects their rights and is recovery‐focused within their communities [16]. Thailand's national mental health policy introduced recovery‐oriented care in 2019 and integrated it into the Department of Mental Health's 2023–2027 operational plan [17, 18]. Key strategies focus on tackling stigma, raising mental health awareness, providing vocational training and building peer support programmes [18]. Personal recovery centres individuals in their care, allowing them to shape meaningful lives based on their own understanding, differing from the symptom‐focused approach of clinical recovery [19]. The CHIME framework, which includes connectedness, hope, identity, meaning in life and empowerment, is central to this process [20]. Le Boutillier et al. suggest that recovery‐oriented practices should support individuals as active participants in their recovery journey, focusing on strengths, respecting individuals' rights, promoting meaningful occupations, fostering community participation, instilling hope and encouraging peer support [21]. However, the concept of personal recovery, originally from Western cultures, requires cultural adaptation in non‐Western countries, particularly in Asia, to ensure its relevance and effectiveness [22, 23, 24].

Thai mental health services are integrated into the broader healthcare system; however, challenges remain, particularly in rural areas where there is a shortage of mental health professionals. Despite the long‐standing emphasis on community‐based care in Thai mental health policy, gaps persist in meeting the mental health needs of remote communities [25, 26]. VHVs are essential assets in bridging the gap between formal healthcare services and the communities they serve [27]. However, it is crucial for researchers to understand the context in which VHVs work to ensure that studies are relevant and acceptable to their practices.

The initial qualitative study offered valuable insights into the practices and roles of VHVs in mental healthcare in Thai rural communities [28]. The findings indicated limited engagement in mental health services, with a predominant emphasis on biomedical approaches. Participants attributed these limitations to low levels of mental health awareness and literacy, and inadequate training [28]. Strengthening VHVs' practices to become more recovery‐oriented is crucial for addressing the needs of individuals with mental health challenges in rural areas, particularly by aligning their roles with recovery‐oriented policies. Acknowledging that VHVs are already required to perform a wide range of tasks, it is essential to understand which areas the community finds most important and is willing to prioritise. These findings informed the design of a subsequent Nominal Group Technique (NGT) study to prioritise key areas for strengthening VHVs' contributions to mental healthcare [29]. Involving a range of local stakeholders ensures that these priorities are practical, recovery‐focused and relevant to the realities of mental healthcare in rural Thailand.

## 2 Aim

This study aims to engage key stakeholders, including VHVs, individuals with mental health challenges, their caregivers and healthcare professionals (HCPs), to collaboratively identify and prioritise areas for VHVs' role expansion, stigma reduction, training needs and common mental health conditions, thereby enhancing their role in supporting the mental health recovery of individuals with mental health challenges.

## 3 Methods

### 3.1 Study Design

The NGT, a structured and flexible consensus method that integrates quantitative ranking with qualitative evaluation to assess stakeholder priorities, was applied [29, 30]. NGT promotes inclusivity and equal participation, mitigates power imbalances within group settings, and facilitates an in‐depth exploration of findings to prioritise areas for improvement [31]. This method has been effectively employed to identify priorities and inform enhancements in mental health services [32]. The NGT process involved five key stages: (1) introduction and explanation, (2) individual idea generation, (3) structured sharing of ideas through a round‐robin approach, (4) group discussion to clarify ideas, and (5) voting and ranking to determine the most preferred options [31, 33, 34].

### 3.2 Participants and Setting

Three groups of stakeholders were recruited: VHVs, HCPs and individuals with mental health challenges/caregivers from three rural sub‐districts in Chae Hom district in Northern Thailand. Three sub‐districts were chosen to ensure participants' accessibility and attendance. Purposive–convenience sampling methods were used to recruit participants with experience in mental health, ensuring they were relevant to the study's objectives and readily accessible. Eligible participants included registered VHVs from Chae Hom, Ban Sa or Wichet Nakhon sub‐districts who had experience supporting at least one household with individuals experiencing mental health challenges. Individuals with mental health challenges were also eligible if they had been identified by VHVs or HCPs as being in stable condition and residing in one of the three sub‐districts. In addition, family members, friends or caregivers with direct experience of providing care, as well as HCPs working at sub‐district health‐promoting hospitals or the district hospital and supervising VHVs, were included. Participants were excluded if they were under 18 years of age, unable to provide informed consent, or unable to speak, read or write Thai.

### 3.3 Recruitment

An NGT group was designed to include 5–9 participants, ensuring a balance between diverse input and the feasibility of reaching conclusions within a reasonable time frame [35]. To facilitate participant recruitment, the lead researcher collaborated with a local mental health nurse, who served as the study's gatekeeper. The nurse was provided with comprehensive research materials, including eligibility criteria, an information sheet and a summary of the NGT questions and process. She distributed these materials to potential participants, screened eligible individuals, and communicated study details through in‐person interactions, telephone calls and text messages via the Line application. Additionally, the nurse assisted in coordinating suitable times for in‐person NGT sessions and ensured that participants had access to the researcher's contact information for any inquiries.

Three separate NGT groups were formed: one in‐person for service providers (including eight VHVs and one nurse), one in‐person for service users/caregivers (three dyads of individuals with mental health challenges and caregivers) and one online meeting for HCPs (including one psychologist, one public health officer and one nurse).

For the service provider group, six VHVs and three HCPs were initially approached and agreed to participate. However, two of the HCPs later became unavailable, so the nurse invited two additional VHVs to join in their place. The service user/caregivers group successfully included three dyads, each consisting of an individual with mental health challenges and their caregiver. In‐person meetings were prioritised to promote richer interaction and engagement, support group dynamics, and build rapport, particularly among the service user group, and to facilitate participation among lay participants (e.g., VHVs, service users and caregivers) who may face technological challenges. To address limited professional representation in the service providers' group, an online follow‐up NGT meeting was conducted with additional HCPs. The online format was selected to accommodate HCPs' scheduling constraints and to leverage their digital literacy.

All participants had previously been provided with participant information sheets outlining the research, the NGT questions and a group process summary. Participants in the in‐person NGT meetings were asked to sign a written consent form and complete a demographic information sheet before the sessions began.

For the online NGT meeting, 15 eligible HCPs were identified within the study settings. From this pool, the nurse contacted two previously unavailable HCPs and one additional HCP, all of whom agreed to participate. With participants' consent, their contact details were shared with the lead researcher (C.K.), who then provided the information sheet, NGT questions and group meeting outline, along with a secure link to access and sign the digital consent form. The lead researcher and the nurse coordinated with participants to schedule a convenient meeting time. Once consent forms were signed and returned, participants were sent the Zoom meeting link and a demographic information sheet to complete.

### 3.4 Data Collection

Three NGT meetings were conducted between August and October 2024, each lasting between 90 and 140 min. The first two NGT meetings with service providers and service users/caregivers were held in‐person within a meeting room in a mental health clinic within a district hospital, and the final meeting with HCPs was conducted online via Zoom.

The research team (C.K., M.L., A.M. and V.T.) conducted an earlier qualitative study, exploring the mental health practices of VHVs in rural Thai communities [28]. The findings revealed that VHVs' involvement in mental healthcare is limited, primarily focusing on medication management and symptom observation, with little attention given to other aspects of care. Individuals with mental health challenges and caregivers reported receiving inadequate mental health support. Stigma and a lack of training further constrained VHVs' roles. These insights informed the NGT questions, which addressed four areas: expanding VHVs' roles, reducing stigma, identifying training needs and recognising common mental health conditions or symptoms. The role expansion question was tailored to participants' specific roles (see Appendix 1), and the questions were developed in English and translated into Thai by the lead researcher.

All three NGT sessions were mainly facilitated by a native Thai doctoral researcher with a background in mental health nursing, supported by 1–2 co‐facilitators in each group meeting. The co‐facilitators varied across the NGT meetings, based on their availability and the session format. Co‐facilitators' support was tailored to the specific needs of each NGT meeting, with common tasks including assisting participants with writing, distributing materials, ensuring forms were correctly completed, serving refreshments and lunch, and notifying the facilitator of any concerns. In the service provider group, the co‐facilitator was a VHV, with one VHV participant requiring writing assistance. For the service user/caregiver group, the same VHV continued as a co‐facilitator, joined by a registered nurse. Their combined efforts were particularly valuable, as four participants, three individuals with mental health challenges and one caregiver, needed writing support. While these participants were able to write, they expressed discomfort in doing so and were more comfortable having someone else write for them. The co‐facilitator in the HCP group, a doctoral nursing researcher, managed the online platform, handled technical aspects and supported communication during the session.

A summary of the NGT group meeting procedures is provided in Table 1, which also highlights the differences between the in‐person and online meetings. Each group meeting followed the same NGT procedure and script to ensure consistency across sessions, with minor adjustments made as needed. In the HCP meeting, we incorporated ideas generated in the service provider and service user/caregiver meetings to encourage further reflection and build on previous insights. After each meeting, the research team held a debriefing session, engaging in reflective discussions and noting key points in field notes to refine the process for the next meeting. The lead researcher also took detailed notes in both Thai and English. The participants were asked for permission to record the meeting. The audio/video recordings were transcribed verbatim and then translated into English by C.K. The generated ideas and any uncertainties were cross‐checked with a bilingual doctoral researcher in nursing.

**Table 1:** A summary of the NGT group meeting procedures.

NGT stages	Procedures of each stage	Session format		Participant dynamics
		In‐person	Online	
Stage 1: Introduction and explanation	The group session began with an introduction covering the research scope, background and session layout. Ground rules on confidentiality and respect were established. Key findings from a qualitative interview on VHVs' mental health practices were also presented.	Key findings were listed on paper for participants to review.	The shared screen feature was used to present session details and key findings.	As the service user group appeared more reserved during initial interactions, comparatively more time was allocated to informal conversation and rapport‐building than with the other groups. This aimed to help participants feel comfortable and at ease before the structured group meeting.
Stage 2: Silent Generation of Ideas	The lead researcher read each question aloud. Participants had the question printed on A4 paper and were instructed to write down as many ideas as they wanted, doing so independently. Participants were encouraged to keep their ideas brief, using only a few words or a short sentence. Writing assistance was available throughout the session.	Pens and question sheets were provided to participants for writing down their ideas.	Participants were asked to bring their own pens and paper to note ideas. The shared screen feature displayed questions along with ideas generated from the previous two NGT group meetings. Participants could select important ideas and add new ones.	Some caregivers were sceptical about relying on VHVs due to their limited mental health knowledge. The facilitator explained that this was mainly due to insufficient training and encouraged participants to suggest ways to better support VHVs.
Stage 3: Round‐Robin Recording of Ideas	Each participant was invited to share their ideas one at a time, which were recorded on a whiteboard using their exact words. This process continued in rounds, encouraging participants to build on others' ideas until no new ideas were generated.	Ideas were recorded on a physical whiteboard in the meeting room.	The whiteboard feature on Zoom was used to record ideas.	Some participants, especially in the service provider and service user/caregiver groups, initially hesitated to share their ideas due to a lack of confidence. However, with encouragement from the nurse and facilitator, participants gradually became more engaged. Some participants deferred to caregivers. For instance, one participant with mental health challenges felt more comfortable sharing her ideas through her caregiver, ensuring her perspectives were included.
Stage 4: Clarification/Group Discussion	The researcher read each recorded idea aloud, inviting participants to ask for clarifications or details. This stage helped ensure that all ideas were clearly understood. Ideas were clarified with probing questions, which helped participants articulate their thoughts more effectively. Participants were also asked if any duplicate ideas could be combined before moving to the ranking stage, with changes made by group consensus.	The researcher led verbal clarifications with the use of probing questions.	The process remained the same, with participants discussing clarifications verbally through Zoom.	
Stage 5: Voting and Ranking	Participants were invited to select their top five ideas from the whiteboard individually, which they considered most important, and rank them in order of significance from 1 to 5. They assigned five points to their highest‐ranked choice, with scores decreasing to one point for their fifth‐ranked idea. The researcher tallied the total votes, and the top five ideas for each question were presented back to the participants to reflect the group's consensus.	Ranking sheets were provided for each participant to complete individually. Refreshments were offered while participants waited for the scores to be tallied, and lunch was provided after the meeting concluded.	The list of ideas was entered into an online ranking platform (www.mentimeter.com). Participants were instructed to use their mobile phones to access the platform and rank the ideas anonymously. Refreshments and lunch were provided after the meeting concluded, with help from a PPI VHV member.	An HCP expressed uncertainty in using the Mentimeter platform. To support their engagement, they were given an opportunity to explore the tool and practice ranking items during the first round. Once they became familiar with the platform and felt comfortable, the formal ranking process commenced.

### 3.5 Data Analysis

Two types of data were obtained from the NGT meetings: quantitative data from the ranked priorities and qualitative data from the transcripts, which captured the reasoning behind individual ideas and discussions.

#### 3.5.1 Ranked Priorities

To quantify the results of the rankings, we adapted the method from Wallace et al. [36]. Participants' rankings were assigned scores: the top‐ranked outcome received 5 points, the second‐highest 4 points, the third 3 points, the fourth 2 points and the fifth 1 point. These scores were then totalled, and the five highest‐ranked ideas for each question were presented to participants, reflecting the group's overall priorities. In cases where ideas received equal scores, the frequency of votes for each idea was used to determine their ranking. Microsoft Excel was used to list the ideas for each question and calculate the scores, enabling the researchers to determine the top five priorities for each question. After the NGT meetings, the scores were reviewed for accuracy to evaluate the level of agreement among participants.

#### 3.5.2 Qualitative Content Analysis

Anonymised transcripts and written lists of ideas were imported into NVivo 14 for data management and coding. Prioritised ideas and related discussions were analysed to provide context and a comprehensive understanding [37]. The analytic process, following Graneheim and Lundman [38], involved identifying and organising key units of meaning into codes, categories and themes. The lead researcher reviewed the transcripts, field notes and audio recordings multiple times to deepen understanding and capture emerging insights. Initial coding involved identifying meaning units and assigning codes, with prioritised ideas treated as such. To avoid lengthy qualitative descriptions, only common prioritised ideas identified across two or more groups were coded (see Table 4), while uncoded ideas were left uncoloured. These codes were reviewed with the research team (M.L., A.M. and V.T.) and grouped into categories based on similarity. Finally, similar categories were grouped into themes and refined with the team for accuracy and transparency.

### 3.6 Trustworthiness

Trustworthiness was ensured by following the guidelines outlined by Lincoln and Guba [39], which emphasised criteria for trustworthiness: credibility, dependability, confirmability and transferability. Credibility was achieved through data triangulation, including multiple data sources (transcripts, reflexive notes and debriefing) and prolonged engagement with the data. Dependability was maintained by keeping detailed records throughout data collection and through meetings with the research team. Confirmability was supported by regular supervision meetings and member‐checking with multiple researchers experienced in qualitative and mental health research, ensuring objectivity and reducing bias. Transferability was enhanced through purposive sampling and by providing detailed descriptions of the research context to facilitate applicability to other settings.

### 3.7 Ethics Consideration

Ethical approval for this study was obtained from King's College London Research Ethics Committee (Reference Number: RESCM‐23/24‐36445) before the commencement of the research. Additionally, local permissions for data collection were granted by the Lampang Provincial Public Health Office (Reference Number: LP0033/4767). The study adhered to the Helsinki Guidelines to ensure compliance with standard research ethics [40].

## 4 Results

### 4.1 Participants

Eighteen participants attended one of three NGT groups (see Table 2 for a summary of participant characteristics). The participants included eight VHVs, four HCPs and three dyads (each consisting of an individual with mental health challenges and their caregiver). VHVs' experience ranged from 2 months to 28 years, with an average of 12 years. Their ages ranged from 38 to 62 years. The HCPs were registered nurses, public health officers and psychologists and had worked at their current locations for between 3 and 39 years, averaging 19 years. The caregivers were family members of individuals with mental health challenges, including husbands, daughters and mothers. Their caregiving experience spanned from 10 to 20 years, with an average of 16 years. The individuals with mental health challenges were all diagnosed with schizophrenia, with the duration of their diagnoses ranging from 10 to 18 years, averaging 14 years.

**Table 2:** Participant characteristics (*N* = 18).

Characteristics	Service provider group (*n* = 9)	Service user/caregiver group (*n* = 6)	Healthcare professional group (*n* = 3)
Participant roles in the study			
VHV			
VHV deputy head	1	N/A	N/A
VHV member	7	N/A	N/A
Healthcare professional			
Registered nurse	1	N/A	1
Public health officer	—	N/A	1
Psychologist	—	N/A	1
Caregivers	N/A	3	N/A
Individual with mental health challenges	N/A	3	N/A
Sex			
Female	7	5	1
Male	2	1	2
Mean age (years)	48	45	42
Educational background			
No formal education	—	1	—
Primary school	1	1	—
High school	2	3	—
Vocational school	4	—	—
Bachelor's degree	1	—	3
Master's degree	1	1	—
Religion			
Buddhism	9	4	3
Christianity	—	1	—
Animism	—	1	—
Ethnicity			
Thai	9	4	3
Lahu	—	2	—

*Note:* N/A = not applicable.

### 4.2 Total Ideas Generated

The three NGT groups generated 94 ideas in response to four questions (see Table 3). Each NGT group produced between 26 and 40 ideas, with some overlapping ideas.

**Table 3:** Total ideas generated for the four questions.

Service provider group	Service user/caregiver group	Healthcare professional group
Question 1: Expanding VHV's role, total ideas generated = 23		
Number of ideas = 8	Number of ideas = 7	Number of ideas = 8
– Encourage patients to engage in daily activities, do household chores, participate in community activities and find hobbies – Find additional jobs and income by identifying the patient's potential and skills – Offer encouragement through conversations – Encourage family members to love and take care of individuals with mental health challenges (some families have negative attitudes towards the patient) – Promote mental health and well‐being by providing knowledge of mental health prevention – Cultivate a public‐minded spirit – Provide counselling and emotional support – Assess stress in both patients and caregivers	– Find jobs for patients to generate income – Establish a VHV centre in the village to report emergencies – Have a VHV group that can provide stress counselling and answer questions about medication – Participate in group activities, and at least some physical movement – Create motivation for patients to have a job and participate in activities – Engage in conversations with patients – Do not view mental health as just a family issue; it's a societal concern requiring support from various sectors	– Low‐intensity counselling – Providing advice, support and encouragement – Promoting community engagement and resilience – Health promotion – Routine home visits to patients – Supporting patient reintegration into the community – Stress assessment – Promoting job opportunities and income generation
Question 2: Reducing stigma, total ideas generated = 26		
Number of ideas = 13	Number of ideas = 8	Number of ideas = 5
– Give patients the opportunity to be part of the community – Encourage patients to work – Engage in casual conversations and greetings to make patients feel they are not alone – Empower and build confidence for patients and their families that they can live with others and encourage them to participate in community activities. – ‐Develop a positive attitude in the community and foster good relationships with patients – Build trust and familiarity – Physical contact (e.g., a pat on the shoulder to encourage) – VHVs visit patients in pairs, with one being an experienced individual (Buddy system) – Have confidence in the treatment process, believing that patients can recover – Nurses build VHVs' confidence in caring for patients and form a support team for patient care. – ‐Show compassion, kindness and love, and treat them well as if they were part of the family – Learn from experienced individuals how to effectively engage with patients – Reduce the dissemination of misinformation on social media	– Do not call them ‘crazy’ – Do not look down on patients – Families should be the main caregivers (if they do not get involved, it is okay even if they speak poorly to us) – Increase VHV and community knowledge and understanding of mental health disorders – Do not put pressure on the patients – Be attentive and encourage patients in tasks they are capable of doing – VHVs should be trained and then share their knowledge with the community – Set an example that mental disorders can be treated, and patients can recover and return to work	– Paired patient visits (Buddy system: experienced person paired with someone less experienced) – Counselling skills – Shaping community attitudes to reduce stigma, particularly by encouraging community leaders or influential individuals to have a proper mindset – Building familiarity and trust through open discussions – Training sessions to enhance knowledge
Question 3: Training needs, total ideas generated = 27		
Number of ideas = 12	Number of ideas = 8	Number of ideas = 7
– Provide training to help VHVs open their hearts and understand patients – Adjust attitudes, thoughts and actions to avoid discrimination against patients – Techniques for approaching patients – Communication skills for interacting with patients – Buddhist approach, emphasising doing good deeds – Training on how to collaborate with other professionals – Develop an application that can locate patients' homes – Provide e‐learning materials on mental health – Encourage VHVs to be eager to learn and focus on practical training through demonstration and return demonstration methods – Patient screening methods and home visit guidelines. – Communicate the risks involved in patient care – Train caregivers to develop positive attitudes and understanding within families towards patients	– Training on how to approach patients – Basic mental healthcare – Provide basic training on each type of mental disorder – Provide knowledge of the severity levels of mental disorders, from mild to severe (not all patients are severe) – Communication skills with patients – Provide guidance on patient care – Have a positive attitude towards patients – Promote career development and future opportunities for patients (discuss and assess the patients' abilities and needs)	– Counselling skills – How to approach patients effectively – Providing ongoing mental health knowledge – Recognising patients as part of the community – Using the ‘Smart VHV’ application with a focus on screening – Understanding symptoms and stages of mental health disorders – Referral process
Question 4: Common mental health conditions, total ideas generated = 18		
Number of ideas = 7	Number of ideas = 5	Number of ideas = 6
– Stress disorder – Depression – Substance addiction (alcohol, drugs) – Hallucinations and auditory delusions – Anxiety disorder – Bipolar disorder – Schizophrenia	– Depression – Hallucinations and auditory delusions – Talking to oneself – Stress – Anxiety	– Schizophrenia: Psychotic states, hallucinations, delusions, paranoia – Substance abuse: amphetamines, alcohol, cannabis – Depression – Anxiety/Panic – Suicidal behaviours – Non‐suicidal self‐injury

### 4.3 Group Priorities

The top five priorities of each group, for each of the four NGT questions, based on the rankings and voting frequencies, are presented in Table 4. Joint rankings were noted for questions on expanding the VHV's role, reducing stigma and training needs, especially in the HCP group.

**Table 4:** Ranking results: Top 5 priorities.

Expanding VHV's role											
Service provider group (*n* = 9)				Service user/caregiver group (*n* = 6)				Healthcare professional group (*n* = 3)			
Rank	Ranked ideas	Total score	Voting frequency	Rank	Ranked ideas	Total score	Voting frequency	Rank	Ranked ideas	Total score	Voting frequency
1	Encourage family members to love and take care of individuals with mental health challenges (Some families have negative attitudes towards patient)	31	9 (100%)	1	Find jobs for patients to generate income	28	6 (100%)	1	Low‐intensity counselling	15	3 (100%)
2	Encourage patients to engage in daily activities, do household chores, participate in community activities, and find hobbies	26	7 (78%)	2	Establish a VHV centre in the village to report emergencies	24	6 (100%)	2	Provide advice, support and encouragement	11	3 (100%)
3	Offer encouragement through conversations	25	7 (78%)	3	Have a VHV group that can provide stress counselling and answer questions about medication	12	4 (67%)	3	Promoting community engagement and building strength in the community	8	3 (100%)
4	Find additional jobs and income by identifying the patient's potential and skills	21	6 (67%)	4	Do not view mental health as just a family issue; it's a societal concern requiring support from various sectors	9	5 (83%)	=4	Health promotion	4	2 (67%)
5	Provide counselling and emotional support	12	6 (67%)	5	Participate in group activities	8	3 (50%)	=4	Support patient reintegration into the community	4	2 (67%)
*Note:* The following colour coding is used to group related ideas into categories: Blue = Emotional and psychological support; Grey = Vocational support; Orange = Family support; Pink = Fostering community reintegration and participation											

**Reducing stigma**											
**Service provider group (*n*** = **9)**				**Service user/caregiver group (*n*** = **6)**				**Healthcare professional group (*n*** = **3)**			
**Rank**	**Ranked ideas**	**Total score**	**Voting frequency**	**Rank**	**Ranked ideas**	**Total score**	**Voting frequency**	**Rank**	**Ranked ideas**	**Total score**	**Voting frequency**
1	Develop a positive attitude in the community and foster good relationships with patients	25	6 (67%)	1	Set an example that mental disorders can be treated, and patients can recover and return to work	19	5 (83%)	1	Paired patient visits (Buddy system: experienced person paired with someone less experienced)	11	3 (100%)
2	Empower and build confidence for patients and their families that they can live with others and encourage them to participate in community activities	22	6 (67%)	2	Families should be the main caregivers (if they do not get involved, it is okay, even if they speak poorly to us)	17	6 (100%)	=2	Counselling skills	10	3 (100%)
3	Engage in casual conversations and greetings to make patients feel they are not alone	18	4 (44%)	3	VHVs should be trained and then share their knowledge with the community	16	4 (67%)	=2	Shaping community attitudes to reduce stigma, particularly by encouraging community leaders or influential individuals to have a proper mindset	10	3 (100%)
4	Encourage patients to work	14	4 (44%)	4	Increase VHV and community knowledge and understanding of mental health disorders	13	4 (67%)	=4	Building familiarity and trust through open discussions	7	3 (100%)
5	VHVs visit patients in pairs, with one being an experienced individual (Buddy system)	12	6 (67%)	5	Do not call them ‘crazy’	12	3 (50%)	=4	Training sessions to enhance knowledge	7	3 (100%)
*Note:* The following colour coding is used to group related ideas into categories: Blue = Shifting attitudes and fostering empowerment; Pink = Promoting mental health literacy and awareness; Grey = Buddy system											

**Training needs**											
**Service provider group (*n*** = **9)**				**Service user/caregiver group (*n*** = **6)**				**Healthcare professional group (*n*** = **3)**			
**Rank**	**Ranked ideas**	**Total score**	**Voting frequency**	**Rank**	**Ranked ideas**	**Total score**	**Voting frequency**	**Rank**	**Ranked ideas**	**Total score**	**Voting frequency**
1	Adjust attitudes, thoughts and actions to avoid discrimination against patients	34	7 (78%)	1	Provide knowledge of the severity levels of mental disorders, from mild to severe (not all patients are severe)	24	5 (83%)	1	Counselling skills	13	3 (100%)
2	Techniques for approaching patients	26	7 (78%)	2	Promote career development and future opportunities for patients (discuss and assess the patients' abilities and needs)	13	5 (83%)	2	How to approach patients effectively	10	3 (100%)
3	Provide training to help VHVs open their hearts and understand patients	21	6 (67%)	3	Basic mental healthcare	12	5 (83%)	3	Providing ongoing mental health knowledge	7	3 (100%)
4	Train on how to collaborate with other professionals	12	6 (67%)	4	Communication skills with patients	12	4 (67%)	=4	Recognising patients as part of the community	6	2 (67%)
5	Communication skills for interacting with patients	12	3 (33%)	5	Provide basic training on each type of mental disorder	11	4 (67%)	=4	Using the ‘Smart VHV’ application with a focus on screening	6	2 (67%)
*Note:* The following colour coding is used to group related ideas into categories: Blue = Communication and engagement skills; Pink = Stigma reduction‐related training; Grey = Mental health awareness education											

**Common mental health conditions**											
**Service provider group (*n*** = **9)**				**Service user/caregiver group (*n*** = **6)**				**Healthcare professional group (*n*** = **3)**			
**Rank**	**Ranked ideas**	**Total score**	**Voting frequency**	**Rank**	**Ranked ideas**	**Total score**	**Voting frequency**	**Rank**	**Ranked ideas**	**Total score**	**Voting frequency**
1	Depression	39	9 (100%)	1	Hallucinations and auditory delusions	22	6 (100%)	1	Schizophrenia: Psychotic states, hallucinations, delusions, paranoia	14	3 (100%)
2	Stress disorder	31	7 (78%)	2	Depression	20	6 (100%)	=2	Substance abuse: amphetamines, alcohol, cannabis	9	3 (100%)
3	Substance addiction (alcohol, drugs)	25	8 (89%)	3	Talking to oneself	16	6 (100%)	=2	Depression	9	3 (100%)
4	Hallucinations and auditory delusions	12	6 (67%)	4	Stress	14	6 (100%)	4	Anxiety/Panic	8	3 (100%)
5	Schizophrenia	11	5 (56%)	5	Anxiety	13	6 (100%)	5	Suicidal behaviours	3	1 (33%)
*Note:* The following colour coding is used to group related ideas into categories: Blue = Psychosis and schizophrenia; Pink = Depression and suicidality; Grey = Stress and anxiety disorders; Orange = Substance use and addiction											

*Note:* ‘*Smart VHV*’ is an application designed for VHVs to submit their monthly performance reports, including health screening and assessment, and also serves as an educational platform for them.

### 4.4 Themes

An analysis of the common top priorities identified for each NGT question has been organised under four key themes, aligning with the NGT question topics. The coding framework overview is presented in Appendix 2.

#### 4.4.1 VHV's Mental Health Recovery Role Enhancement

This theme emphasised the enhanced and expanded role of mental health recovery as identified by service providers and HCPs and the support needs articulated by service users/caregivers. The key areas included: *(1) Vocational support*, *(2) Family support, (3) Emotional and psychological support*, and *(4) Fostering community reintegration and participation*.

##### 4.4.1.1 Vocational Support

Vocational support for helping individuals with mental health challenges find jobs and generate income was prioritised by service users/caregivers (ranked 1st) and service providers (ranked 4th). Service providers suggested that VHVs could play a key role in identifying individuals' skills, potential and interests to match them with suitable work, such as weaving, sewing, gardening or repairing electrical appliances. They also believed that individuals with mental health challenges are capable of working.

While employment was a priority for individuals with mental health challenges, some caregivers were concerned about their ability to maintain a job due to fluctuating motivation and mental health challenges. Nonetheless, caregivers acknowledged that having a job gives individuals a sense of pride and purpose. A caregiver suggested that small, manageable tasks providing a modest income could be more suitable and meaningful than full‐time employment.

*I feel happy. I'm proud to have a job and to earn money.*

03Individual with mental health challenges

##### 4.4.1.2 Family Support

The crucial role of family support in the care of individuals with mental health challenges was emphasised by both service providers (ranked 1st) and service users/caregivers (ranked 4th). Service providers suggested that VHVs should help families foster love and understanding, especially when family members show reluctance or negative attitudes. Some VHVs shared a case where a father's neglect contributed to the suicide of his child with depression:

*His son was hungry, but instead of finding food for them, the father went off to farm and said, “If you're going to die, just die.”*

07VHV

VHVs highlighted the need for better understanding and communication from family members, suggesting that caregivers also need proper training. For instance, they felt VHVs can encourage families to approach those with mental health challenges with understanding, be patient and gentle with their emotions, avoid harsh words that could lead to harm, and offer ongoing support through regular VHV visits. Service providers also stressed that when family support is lacking, VHVs must step in, using community resources to provide care, such as coordinating food donations for those in need.

A caregiver further emphasised the societal responsibility in supporting individuals with mental health challenges, noting that mental health should not be seen as solely a family issue. Communities should also take an active role, especially when family members neglect the individual, to prevent treatment delays and ensure proper care is provided.

##### 4.4.1.3 Emotional and Psychological Support

All groups agreed that providing emotional support and basic psychological care were key areas to enhance VHVs' role. Caregivers emphasised the importance of VHVs engaging in conversation, as many individuals were treated with disrespect and heard demeaning comments. This view aligned with service providers and HCPs, who also highlighted the need for VHVs to provide emotional encouragement and basic counselling.

This was echoed by an HCP, who suggested that VHVs could provide low‐intensity counselling (ranked 1st by HCPs). This approach is seen as feasible by other HCPs, as it could be integrated into VHVs' regular home visits, where they already assess physical, mental and social aspects. However, it was acknowledged that adequate training is essential for VHVs to implement this successfully.

##### 4.4.1.4 Fostering Community Reintegration and Participation

All NGT groups highlighted the important role of VHVs in supporting individuals with mental health challenges to reintegrate into the community and participate in activities. Concerns about individuals becoming idle and the need to encourage participation in community activities were expressed by service providers (ranked 2nd) and service users/caregivers (ranked 5th). They noted that without VHV support, inactivity could worsen their condition. For example, one VHV shared how an individual with mental health challenges, encouraged by VHVs, helped at a temple by sweeping the grounds or assisting monks during their alms round.

The HCP group noted that reintegration situations varied by location. In communities with greater mental health understanding, VHVs felt more confident providing care. However, in areas with less awareness, there was a fear of relapse or potential violence. To address this, they suggested that VHVs should enhance understanding and community support for individuals with mental health challenges. The goal is to equip VHVs with the necessary knowledge and tools to assess individuals, knowing when to intervene and seek support from HCPs.

An HCP from the service provider group emphasised the importance of a well‐coordinated reintegration strategy, explaining that community acceptance, particularly from leaders, such as the village headman, is essential. Other HCPs agreed, stressing that active community participation and collaboration among community leaders, VHVs and governing officers are crucial for successful and sustainable reintegration (ranked 3rd and 4th).

*If a person returns to the community and is seen as an outsider, it can be challenging. If the community doesn't accept or understand, especially the community leaders or VHVs, residents might become even more fearful.*

03HCP

#### 4.4.2 Stigma Reduction

This theme included prioritised strategies to tackle stigma, namely: *(1) Shifting attitudes and fostering empowerment, (2) Buddy system and (3) Promoting mental health literacy and awareness*.

##### 4.4.2.1 Shifting Attitude and Fostering Empowerment

Shifting attitude and fostering empowerment were key to breaking down the stigma surrounding mental health across all groups. Stigma remains a major concern, with individuals experiencing mental health challenges expressing frustration about being labelled ‘*crazy’*.

*They say I'm crazy…. It really sticks with me.*

01Individual with mental health challenges

These insights underscored the need for VHVs to address stigma by fostering a more accepting community and promoting understanding (ranked 1st by service providers and 2nd by HCPs). Service providers and HCPs emphasised educating both the community and VHVs that individuals with mental health challenges are ‘*just like us’ (06VHV)* and should not be seen as dangerous. Increasing VHVs' exposure to mental health work is crucial for building familiarity and trust. Many noted that regular interaction and compassionate communication have positively impacted reducing fear and misconceptions. HCPs stressed the importance of community leaders in shaping public attitudes, as their support is key to the long‐term success of stigma reduction efforts.

Service users/caregivers mentioned that empowering patients and their families by instilling confidence in recovery and in their ability to reconnect with others also contributes to reducing stigma. Real‐life examples of recovery (ranked 1st by service users/caregivers) can inspire hope and foster greater acceptance in society.

*I might use my mother as an example. Her condition has improved, which should show others that recovery is possible. They can get better … we can at least set an example for others.*

02Caregiver

##### 4.4.2.2 Buddy System

The buddy system, where experienced VHVs or HCPs are paired with less experienced VHVs during visits to individuals with mental health challenges, was considered a crucial method for reducing stigma by both HCPs (ranked 1st) and service providers (ranked 5th). This approach boosted confidence, enhanced their capacities, eased fears and provided hands‐on training. It enabled VHVs to gain practical experience and develop familiarity with individuals with mental health challenges, while also instilling pride in working collaboratively with the staff team.

*They will see that the patients are not dangerous. We show them what to do when certain events happen. We teach them on‐site.*

02HCP

##### 4.4.2.3 Promoting Mental Health Literacy and Awareness

Enhancing mental health knowledge among VHVs and the broader community was crucial for tackling stigma and fostering inclusivity. Across three NGT groups, stigma often arose from a lack of understanding of mental health conditions. One caregiver said that misconceptions and a lack of awareness often led to delayed treatment, worsening the individual's condition. In some communities, individuals with mental health challenges were left untreated because families believed their conditions would only deteriorate or were untreatable.

Raising awareness and improving mental health literacy (ranked 4th by service users/caregivers and HCPs) helped reduce fear, promoted early intervention and led to better outcomes. It also fostered recognition of each individual's unique experience. Additionally, service users/caregivers suggested that trained VHVs should act as community representatives (ranked 3rd), using their knowledge to challenge misconceptions and promote acceptance.

#### 4.4.3 Training Needs

Training priorities included: *(1) Stigma reduction‐related training, (2) Communication and engagement skills and (3) Mental health awareness education*.

##### 4.4.3.1 Stigma Reduction‐Related Training

Both service providers and users/caregivers ranked stigma reduction‐related training as their top priority. Despite being linked to the stigma reduction theme, this reflects the interconnected nature of these priorities. Service users/caregivers emphasised educating people on the varying severity of mental health conditions, noting that not all cases were severe. This helped VHVs feel more confident in supporting individuals with mental health challenges (ranked 1st by service users/caregivers). Training VHVs to develop empathy and openness, recognise individuals as integral members of the community, and adjust their attitudes and actions to prevent discrimination was also deemed essential (ranked 1st and 3rd by service providers and 4th by HCPs).

*I wanted all VHVs to open their minds to mental health patients.*

01VHV

##### 4.4.3.2 Communication and Engagement Skills

Communication and engagement skills were identified as a priority training need across all groups. Service users/caregivers emphasised the importance of training VHVs in how to interact with individuals with mental health challenges (ranked 4th). This included the need for VHVs to speak kindly and use appropriate techniques to better understand and respond to individuals' emotional needs, which could positively influence their condition. VHVs themselves also acknowledged the challenges they faced when communicating with individuals with mental health challenges, stressing the need for training in suitable language and techniques (ranked 2nd and 5th). HCPs corroborated this, noting that VHVs often sought guidance on how to engage with patients effectively. They recommended that communication training include practical sessions alongside theoretical instruction to enhance VHVs' skills (ranked 2nd).

##### 4.4.3.3 Mental Health Awareness Education

Both service users/caregivers and HCPs prioritised several key areas of mental health knowledge training. Service users/caregivers emphasised training in basic mental healthcare techniques (ranked 3rd), including knowing how to deliver mental health or psychological first aid and manage relapses and emergencies before HCP support arrives. This training is believed to enable VHVs to effectively fulfil their role, as they are often the first to report critical issues to HCPs. Moreover, they emphasised training in understanding different types of mental health conditions to provide individualised care (ranked 5th).

HCPs prioritised counselling skills (ranked 1st), recognising VHVs' significant potential in mental health support, though they acknowledged that mastering these skills would take time. They also suggested extending counselling training to parents of children with mental health challenges to enable better support at home. Additionally, HCPs advocated for ongoing mental health training (ranked 3rd), recommending regular refresher courses on mental health conditions, symptoms and assessment techniques to enhance VHVs' community support.

#### 4.4.4 Common Mental Health Conditions

The most commonly observed mental health conditions included psychosis and schizophrenia, depression and suicidality, stress and anxiety disorders, and substance use and addiction.

Psychosis and schizophrenia were frequently reported, with symptoms, including hallucinations, delusions, paranoia and talking to oneself (ranked 1st by HCPs, 1st and 3rd by service users/caregivers, and 4th and 5th by service providers). Depression and suicidality commonly presented as low mood, social withdrawal and suicidal behaviours (ranked 1st by service providers, 2nd by service users/caregivers and 2nd and 5th by HCPs). Stress and anxiety disorders were often linked to daily stress, panic and chronic illness strain (ranked 2nd by service providers, 4th and 5th by service users, and 4th by HCPs). Substance use and addiction raised concerns about drug‐induced psychosis, with amphetamine and alcohol identified as problematic substances (ranked 2nd by HCPs and 3rd by service providers).

*People who take a lot of drugs can get delusional.*

03VHV

## 5 Discussion

This study builds on previous research exploring the roles of VHVs in mental healthcare in rural Thailand, offering new insights into stakeholder‐driven priorities for enhancing their support for mental health recovery [28]. Through NGT, participants generated 94 ideas, highlighting collective concerns and aspirations for a more recovery‐oriented, community‐based mental health approach. Despite varied priorities, four themes emerged: VHVs' mental health recovery role enhancement, stigma reduction, training needs and common mental health conditions.

The categories identified in our findings reflect the core components of the CHIME framework of personal recovery, which includes connectedness (as evidenced in our data, such as community reintegration and family support), hope and optimism (e.g., participant calls for positive attitudes), identity (e.g., supporting individuals in reclaiming community roles), meaning in life (e.g., suggestions for vocational support), and empowerment (e.g., addressing stigma, improving mental health literacy and equipping VHVs with recovery‐supportive skills) [20]. These examples, directly derived from stakeholder input, demonstrate how community perspectives can inform the role of VHVs in supporting recovery‐oriented mental healthcare, while also supporting national policy directions in Thailand [18] and international guidance from WHO [16].

To enable VHVs to deliver recovery‐oriented care, they must operate within pro‐recovery, non‐stigmatising environments to optimise the impact of recovery interventions [41]. Stigma remains a significant barrier to recovery, affecting both access to care and individual outcomes [42]. Despite policy support for recovery‐oriented practices with a focus on destigmatisation [16, 18], stigma persists across multiple levels [43]. This disconnect between policy and practice was evident in service user accounts, with some expressing distress at being labelled as ‘crazy’. Stigma by association was also observed, as family members occasionally made jokes that reinforced harmful stereotypes. Public stigma, where individuals are viewed as dangerous, also emerged as a major concern [43]. Stigma affects not only Thailand but also other Asian countries, such as Singapore, Japan, Nepal and the Philippines, where individuals with mental health challenges are often perceived as weak, dangerous and unpredictable and labelled with stigmatising terms [44]. Addressing stigma is crucial for individual recovery and fostering a supportive community environment. Participants prioritised attitude shifts at all levels—within families, among VHVs and across the wider community. They highlighted the importance of involving community leaders, including local and political figures, alongside VHVs, to drive change. These leaders are crucial in transforming attitudes towards mental health, reintegration and community engagement. At the system level, collaboration among key local actors, public sector partnerships and the use of monitoring tools are vital for effective implementation and sustained success [45, 46].

Mental health education, especially about the varying severity of conditions, is crucial for challenging misconceptions and fostering understanding. Service users and caregivers highlighted the importance of demonstrating that recovery is possible, aligning with the ideas of involving individuals with lived experience to instil hope, support others and promote a more positive image of individuals with mental health challenges [21]. Anti‐stigma interventions in Asian and LMICs, such as SMART, RESHAPE and TC‐GLOBAL, focus on enhancing mental health literacy and implementing interaction‐based approaches. These strategies are similar to successful programmes in high‐income countries, although their long‐term impact requires further study [47]. This study also identified common mental health concerns in the community, including psychosis, schizophrenia, depression and stress. These insights point to key areas where targeted education and awareness‐raising could enhance symptom recognition, improve care and reduce stigma. Addressing stigma through education, the inclusion of individuals with lived experience, and community engagement is, therefore, a critical first step in enabling VHVs to deliver effective, recovery‐oriented care.

Cultural context plays a vital role in shaping recovery‐oriented care. In Asian societies such as Thailand, family, religion and social support are central to the recovery experience [24, 48]. Given that many recovery approaches originate from individualistic cultures [20], adapting these approaches to reflect local values is essential. In Thailand, for example, Buddhism significantly influences beliefs about mental health, suffering and healing. Religious practices such as mindfulness, compassion and acceptance are often integrated into daily life and can support recovery. Locally developed, culturally sensitive interventions such as Buddhist counselling and mindfulness meditation have shown positive outcomes for individuals experiencing anxiety and depression [49, 50, 51].

Family involvement is also crucial, particularly in cultures where families serve as the primary support system [48, 52]. Our study supports expanding assistance for family caregivers, a top priority identified by VHVs. By encouraging compassionate and informed care, VHVs can help prevent serious consequences and improve home care. Research from China [53] highlights the burdens on caregivers of people with schizophrenia due to inadequate support, emphasising the need for expanded services, public awareness and proper caregiver training. This can improve long‐term outcomes for individuals with schizophrenia [54]. Limited understanding of mental health conditions among families may also lead to doubts about their loved ones' ability to maintain employment. Caregivers' concerns may stem from relationship challenges, which can worsen without adequate support [55]. This underscores the need for better understanding and support, with VHVs providing essential caregiver skills and resources.

In addition, expanding VHVs' roles to include job support emerged as a top priority among service users and caregivers, underscoring the critical role of employment in the recovery process. Employment not only provides financial benefits but also enhances self‐esteem, pride and a sense of identity, which are vital to individuals' well‐being [56]. In parallel, HCPs emphasised the importance of equipping VHVs with low‐intensity counselling skills. Evidence suggests that lay health worker‐delivered counselling can contribute positively to mental health recovery [57]. However, to ensure effectiveness, such training must include role‐playing, skill rehearsal and structured supervision. These components are essential for building VHVs' competence and confidence. Without adequate support, VHVs may feel overwhelmed by the expansion of their responsibilities, perceiving it not as a meaningful redistribution of tasks (task‐shifting) but rather as an unfair transfer of duties without proper training or resources (task‐dumping) [58].

### 5.1 Implications for Future Practice and Policy

The study highlights key opportunities to strengthen community mental health systems by enhancing the roles of VHVs, particularly in remote areas. The findings offer practical insights applicable to similar LMIC contexts, especially within Asia. A key priority is addressing stigma through context‐specific, co‐designed interventions that reflect local realities. Future efforts should prioritise the development of targeted training programmes, stigma reduction strategies and the expansion of VHVs' roles in community‐based mental health recovery. Given their deep understanding of local needs and resources, VHVs are uniquely positioned to support recovery, especially when equipped with appropriate training and support. To foster more supportive environments, it is essential to implement awareness campaigns and community engagement initiatives that challenge misconceptions about mental health. Strengthening partnerships with families and community leaders will further enhance the effectiveness and sustainability of interventions. Additionally, investing in ongoing training and structured supervision by mental health professionals will help ensure that VHVs are equipped with the necessary skills and confidence to fulfil their roles. Clear policy guidance is also needed to define VHVs' mental health responsibilities, ensuring alignment with recovery‐oriented principles and long‐term system strengthening.

### 5.2 Strengths and Limitations

The NGT approach enabled the gathering of various stakeholders' perspectives and empowered participants to voice their priority preferences, ensuring equal participation and addressing local needs in under‐researched areas. However, this study has some limitations. The sample was small and drawn from a single province, with all individuals with mental health challenges diagnosed with schizophrenia. This may limit the generalisability of the findings to other regions with different geographical, cultural and contextual factors or diagnoses [59]. While including service user dyads provided valuable insights into their shared experiences, their perspectives may have influenced one another. Nevertheless, this interaction enriched the understanding of the support systems and challenges they faced. Despite efforts to include a broad range of stakeholders, future studies could benefit from including other key figures, such as community leaders and local authorities, to gain insights into feasibility and policy alignment and foster collaborative efforts. Participation from HCPs was limited, with only three attending the third meeting due to competing clinical and professional commitments. During the ranking stage, concerns were raised regarding the potential impact of small group sizes on the validity of the results, as individual rankings could disproportionately influence outcomes [60]. To mitigate this, participants were encouraged to carefully review and rank ideas based on their considered preferences. Lastly, language translation may have resulted in some loss of meaning; however, a bilingual doctoral researcher cross‐checked the translation, and both Thai and English transcripts were analysed to ensure accuracy and capture nuances.

## 6 Conclusion

The findings highlight key priorities for enhancing VHVs' roles in mental health recovery in rural Thai communities, including vocational support, family support, community reintegration and counselling skills. Addressing stigma is essential and can be achieved through increased awareness, literacy and education at all levels. Future research should focus on stigma reduction interventions and training to support VHVs' practice to better align with recovery principles.

## Author Contributions

**Chonmanan Khanthavudh:** conceptualisation, methodology, formal analysis, investigation, data curation, writing – original draft preparation, writing – reviewing and editing, visualisation. **Annmarie Grealish:** conceptualisation, methodology, supervision, validation, writing – original draft preparation, writing – reviewing and editing, visualisation. **Vasiliki Tzouvara:** conceptualisation, methodology, supervision, validation, writing – original draft preparation, writing – reviewing and editing, visualisation. **Mary Leamy:** conceptualisation, methodology, supervision, validation, writing – original draft preparation, writing – reviewing and editing, visualisation.

## Ethics Statement

Ethical approval for this study was obtained from the King's College London Research Ethics Committee (Reference Number: RESCM‐23/24‐36445), before the commencement of the research. Additionally, local permissions for data collection were granted by the Lampang Provincial Public Health Office (Reference Number: LP0033/4767). The study adhered to the Helsinki Guidelines to ensure compliance with standard research ethics [40].

## Conflicts of Interest

The authors declare no conflicts of interest.

## Data Availability Statement

The data that support the findings of this study are available on request from the corresponding author. The data are not publicly available due to privacy or ethical restrictions.

## Supporting information

Appendix 1. NGT questions used in this study.

Appendix 2. Coding framework overview_clean.
