# Supplementary material for: Identifying Priorities for Enhancing Village Health Volunteer's Mental Health Recovery Practices in Thai Rural Communities: A Nominal Group Technique Study
Source: Health Expect. 2025 Oct 3;28(5):e70455. doi: 10.1111/hex.70455 (PMC12492073; doi:10.1111/hex.70455)
Supplement: Supplementary file 1 — Appendix 1. NGT questions used in this study. [file HEX-28-e70455-s001.docx]

**Appendix 1.** NGT questions used in this study

| **Questions** | **Service provider and Healthcare professional groups** | **Service user/caregiver group** |
| --- | --- | --- |
| **1. Expanding VHV’s role** | *To what extent do you think VHVs could expand their role in mental health beyond just focusing on medication and symptoms?* | *What are the main needs of people with mental health challenges in the community that VHVs can address to support their recovery?* |
| **2.Reducing stigma** | *What can be done to reduce the negative attitudes that VHVs have towards people with mental health challenges?* | |
| **3.Training needs** | *What are key training priorities for VHVs to improve their mental health practice?* | |
| **4.Common mental health conditions/diagnoses/symptoms*** | *What common mental health conditions, diagnoses, or symptoms do you encounter?* | |

***** Due to the diverse backgrounds of participants, the fourth question focused on common mental health conditions and diagnoses, offering symptoms as an alternative if participants were unsure of the specific condition.
