# Supplementary material for: Identifying Priorities for Enhancing Village Health Volunteer's Mental Health Recovery Practices in Thai Rural Communities: A Nominal Group Technique Study
Source: Health Expect. 2025 Oct 3;28(5):e70455. doi: 10.1111/hex.70455 (PMC12492073; doi:10.1111/hex.70455)
Supplement: Supplementary file 2 — Appendix 2. Coding framework overview_clean. [file HEX-28-e70455-s002.docx]

**Appendix** **2. Coding framework overview**

| **Themes** | **Categories** | **Prioritised ideas as Example codes** | **Example quotes** |
| --- | --- | --- | --- |
| VHV’s mental health recovery role  enhancement | Vocational support | Find additional jobs and income by identifying the patient’s potential and skills | 04VHV: Helping them find an additional income, enhancing their capabilities. It's about discovering what they are able to do. We can find tasks for them that they are capable of. For example, if they are at home, a woman could learn to crochet or knit. |
|  |  | Find jobs for patients to generate income |  |
|  | Family support | Encourage family members to love and take care of individual with mental health challenges. (Some families have negative attitudes towards patient) | 07VHV: There are cases of depression where the family doesn't understand. Family plays such an important role. Like in one suicide case, the father didn't take care of his son. His son was hungry, but instead of finding food for them, the father went off to farm and said, "If you're going to die, just die." So, the son ended up hanging themselves. |
|  |  | Do not view mental health as just a family issue; it's a societal concern requiring support from various sectors |  |
|  | Emotional and psychological support | Offer encouragement through conversations | 02HCP: Low-intensity counselling requires skills that VHVs can learn. For example, they can make home visits, which is part of their routine anyway. These visits could include basic counselling skills and talking to patients effectively. These VHVs already handle hypertension and diabetes; adding this component wouldn’t be too difficult. That’s what I’d like to see happen—it’s entirely doable. |
|  |  | Provide counselling and emotional support |  |
|  |  | Have a VHV group that can provide stress counselling |  |
|  |  | Low-intensity counselling |  |
|  |  | Provide advice, support, and encouragement |  |
|  | Fostering community reintegration and participation | Encourage patients to engage in daily activities, do household chores | 03HCP: I’d like to go back to the topic of reintegration into the community. I think this is very important. If a patient returns to the community and is seen as an outsider, it can be challenging. If the community doesn’t accept or understand, especially the community leaders or VHVs, the residents might become even more fearful. |
|  |  | Participate in group activities |  |
|  |  | Promoting community engagement and building strength in the community |  |
|  |  | Support patient reintegration into the community |  |
| Stigma reduction | Shifting attitudes and fostering empowerment | Develop a positive attitude in the community and foster good relationships with patients | 06VHV: It’s about changing the community's attitude towards patients. Sometimes, people say things like, “This person is a mental health patient; they’re not safe to be around; they’re scary.” We should help them understand that patients are just people like us. |
|  |  | Shaping community attitudes to reduce stigma, particularly by encouraging community leaders or influential individuals to have a proper mindset |  |
|  |  | Engage in casual conversations and greetings to make patients feel they are not alone |  |
|  |  | Building familiarity and trust through open discussions |  |
|  |  | Empower and build confidence for patients and their families that they can live with others and encourage them to participate in community activities |  |
|  |  | Set an example that mental disorders can be treated, and patients can recover and return to work |  |
|  |  | Do not call them "crazy" |  |
|  | Buddy system | VHVs visit patients in pairs, with one being an experienced individual (Buddy system) | 02HCP: If we want VHVs to reduce their stigma toward patients, it’s really about reducing their fear, as I mentioned earlier. One approach I’ve thought of is conducting patient visits in pairs, like a buddy system. Pair up someone with experience and someone without, or with staff members. |
|  |  | Paired patient visits |  |
|  | Promoting mental health literacy and awareness | VHVs should be trained and then share their knowledge with the community | 02HCP: I think it’s like this: If the VHVs have more knowledge, they will be less afraid of the patients because mental health issues are something they are not familiar with. They don’t know when something dangerous might happen. The more they learn, the less fear they’ll have about the patients. |
|  |  | Increase VHV and community knowledge and understanding of mental health disorders. |  |
|  |  | Training sessions to enhance knowledge |  |
| Training needs | Stigma reduction-related training | Adjust attitudes, thoughts, and actions to avoid discrimination against patients | 01Individual with mental health challenges: I don’t want them to be afraid, have a positive attitude and don’t think that all patients are bad. It’s not necessary that everyone with mental health issues has to be aggressive. |
|  |  | Provide training to help VHVs open their hearts and understand patients |  |
|  |  | Provide knowledge on the severity levels of mental disorders, from mild to severe. |  |
|  |  | Recognising patients as part of the community |  |
|  | Communication and engagement skills | Techniques for approaching patients | 01Individual with mental health challenges: To know how to approach patients and speak kindly. Not to look down on us and to speak nicely to us. So, they can understand the patients’ feelings. |
|  |  | Communication skills for interacting with patients |  |
|  |  | Communication skills with patients |  |
|  |  | How to approach patients effectively |  |
|  |  | Counselling skills |  |
|  | Mental health awareness education | Basic mental health care | 02Caregiver: To train on the different types of mental disorders. |
|  |  | Provide basic training on each type of mental disorder |  |
|  |  | Providing ongoing mental health knowledge |  |
| Common mental health conditions | Psychosis and schizophrenia | Hallucinations and auditory delusions | 02HCP: I think the most common one is psychosis, which includes hallucinations—like seeing or hearing things—and delusions, such as paranoia or false beliefs. |
|  |  | Schizophrenia: Psychotic states, hallucinations, delusions, paranoia |  |
|  |  | Talking to oneself |  |
|  | Depression and suicidality | Depression | 02Caregiver: Mostly in the village and sometimes at church, I see people with depression. |
|  |  | Suicidal behaviours |  |
|  | Stress and anxiety disorders | Stress disorders | 04VHV: From what I've seen, it's mostly stress-related disorders. |
|  |  | Anxiety/Panic |  |
|  | Substance use and addiction | Substance addiction (alcohol, drugs) | 02HCP: The most severe cases requiring treatment are, first, amphetamines, and second, alcohol. |
|  |  | Substance abuse: amphetamines, alcohol, cannabis |  |

**Note:** HCP = Healthcare professional, VHV = Village health volunteer
